# Supplementary material for: Elevated hematopoietic stem cell frequency in mouse alveolar bone marrow
Source: Stem Cell Reports. 2024 Dec 12;20(1):102374. doi: 10.1016/j.stemcr.2024.11.004 (PMC11784484; doi:10.1016/j.stemcr.2024.11.004)
Supplement: Document S1. Figures S1–S4 [file mmc1.pdf]

Stem Cell Reports, Volume *20*

Supplemental Information

Elevated hematopoietic stem cell frequency in mouse alveolar bone marrow

Kouta Niizuma, Satoru Morikawa, Eric Gars, Jinyi Xiang, Tomoko Matsubara-Takahashi, Rei Saito, Eri Takematsu, Yuting Wang, Haojun Xu, Arata Wakimoto, Tze Kai Tan, Yoshiaki Kubota, Charles K.F. Chan, Irving L. Weissman, Taneaki Nakagawa, Adam C. Wilkinson, Hiromitsu Nakauchi, and Ryo Yamamoto

Figure S1

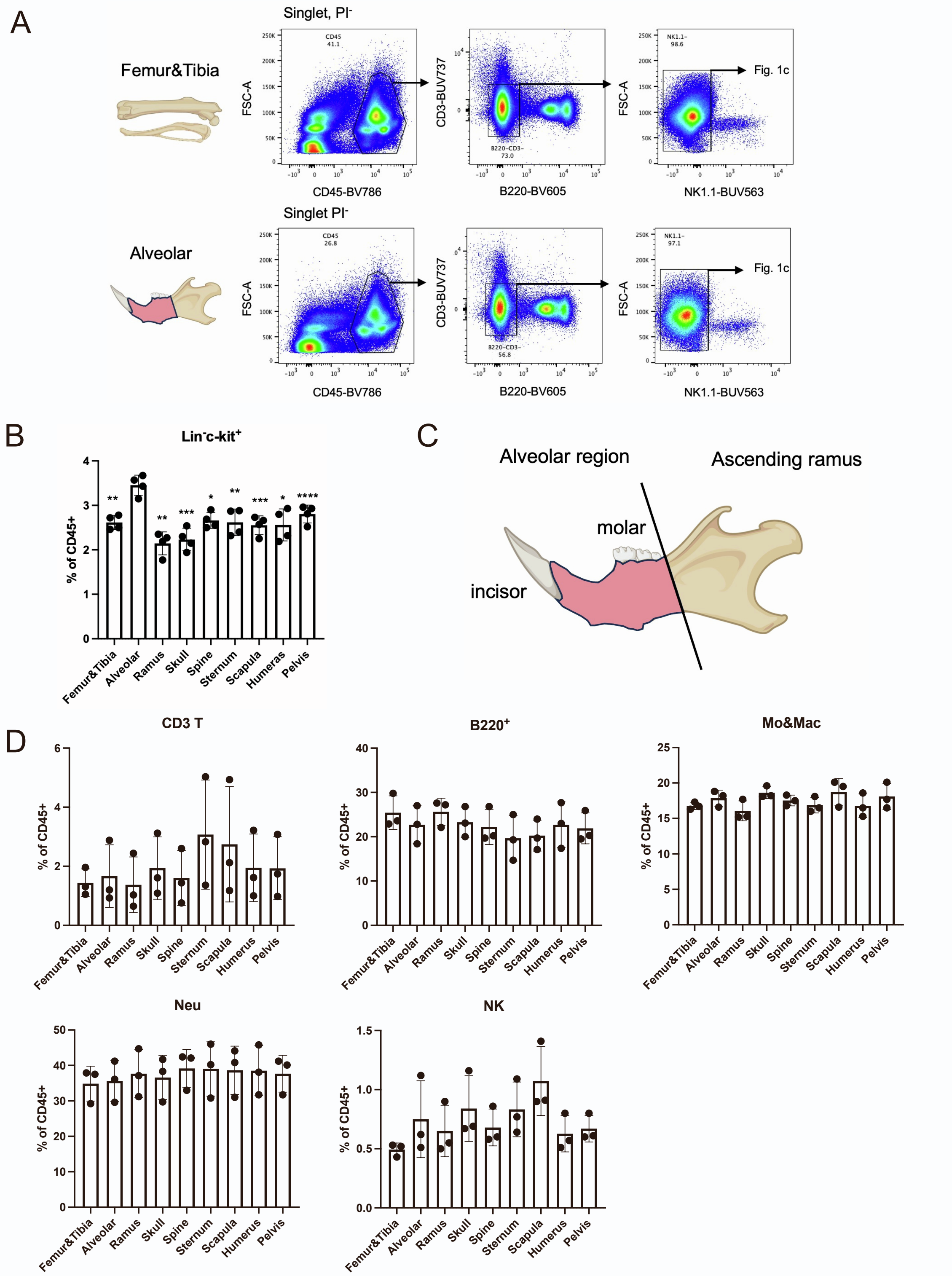

Figure S1. Enhanced Frequency of Functional HSCs in Alveolar Bone Marrow Compared to Combined Femur, Tibia, and Pelvis Bone Marrow.

(A) Representative flow cytometry plots displaying the immunophenotypic analysis of HSCs in ftp-BM (combined femur, tibia, and pelvis bone marrow) and al-BM (alveolar bone marrow) for gating out Lin^+ cells.

(B) Frequency of phenotypic hematopoietic stem/progenitor cell populations ($Lin^-c\text{-}Kit^+$) within $CD45^+$ BM cells from nine different skeletal sites, highlighting the elevated frequency in al-BM. Data are presented as mean ± SEM. Statistical significance was determined using a paired two-tailed Student's t-test for each comparison: $^*p < 0.05$, $^{**}p < 0.01$, $^{***}p < 0.001$, $^{****}p < 0.0001$. Data are presented as mean ± SEM from four independent experiments ($n = 4$).

(C) Schematic illustration of the mandibular bone regions, including the alveolar region and ascending ramus, used for the isolation of al-BM.

(D) The graphs display the frequency of different immune cell populations within the $CD45^+$ cell compartment across nine different skeletal sites. The immune cell populations analyzed include: $CD3^+$ T cells, $B220^+$ cells, Mo/Mac (monocytes/macrophages, $CD11b^+Ly6G^-$), Neu (neutrophils, $CD11b^+Ly6G^+$), and NK (natural killer, $NK1.1^+$). The data are presented as mean ± SEM, and no statistically significant differences were observed in the frequencies of these immune cell populations among the different bone marrow sites. Data are presented as mean ± SEM from three independent experiments ($n = 3$).

Figure S2

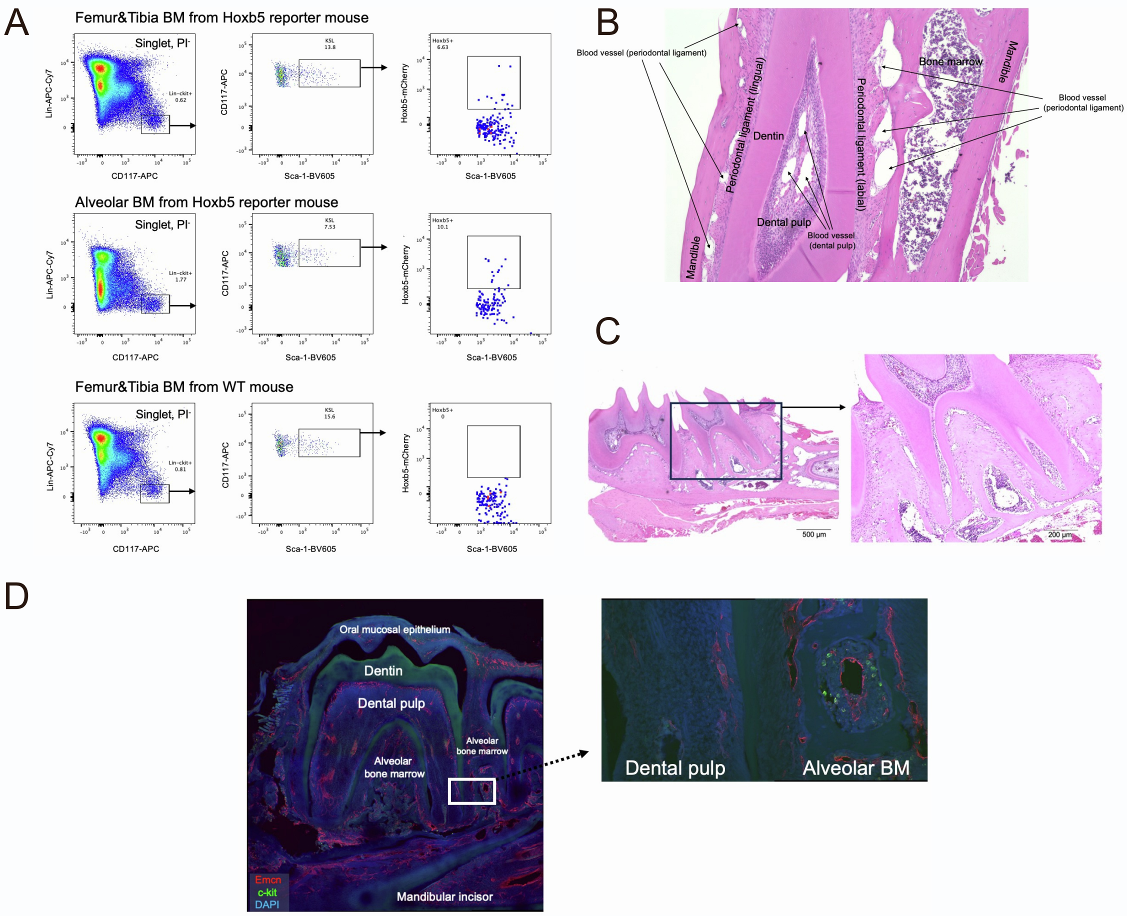

Figure S2 Identification and Histological Analysis of Hematopoietic Stem/Progenitor Cells in Alveolar and Femur/Tibia Bone Marrow

(A) Identification of Long-term Hematopoietic Stem Cells Using Hoxb5 Reporter Mouse. Representative flow cytometry plots displaying the gating strategy for identifying KSL and Hoxb5^{-}mCherry^{+} KSL populations. Data are shown for femur/tibia bone marrow (ft-BM) and alveolar bone marrow (al-BM) from Hoxb5 reporter mice, as well as femur/tibia bone marrow (ft-BM) from wild-type (WT) mice as a control.

(B) Representative H&E-stained section of the mandible, showing various structures including the dentin, periodontal ligament, dental pulp, and bone marrow. The periodontal ligament and dental pulp contain blood vessels, while the bone marrow shows distinct hematopoietic cell populations.

(C) Overview of the mandible section with detailed inset. The left image shows a low magnification view of the mandible section, highlighting the regions of interest. The right image is a higher magnification view of the boxed area, detailing the alveolar bone marrow and adjacent structures. Scale bars: 500 μm (left) and 200 μm (right).

(D) Immunohistochemical Analysis of Alveolar Bone Marrow and Dental Pulp. Composite image of a mandibular section from a P18 mouse stained for Emcn (red), c-Kit (green), and DAPI (blue). The left panel shows a low magnification view of the mandibular incisor region, while the right panel shows a higher magnification view of the alveolar BM and dental pulp.

Figure S3

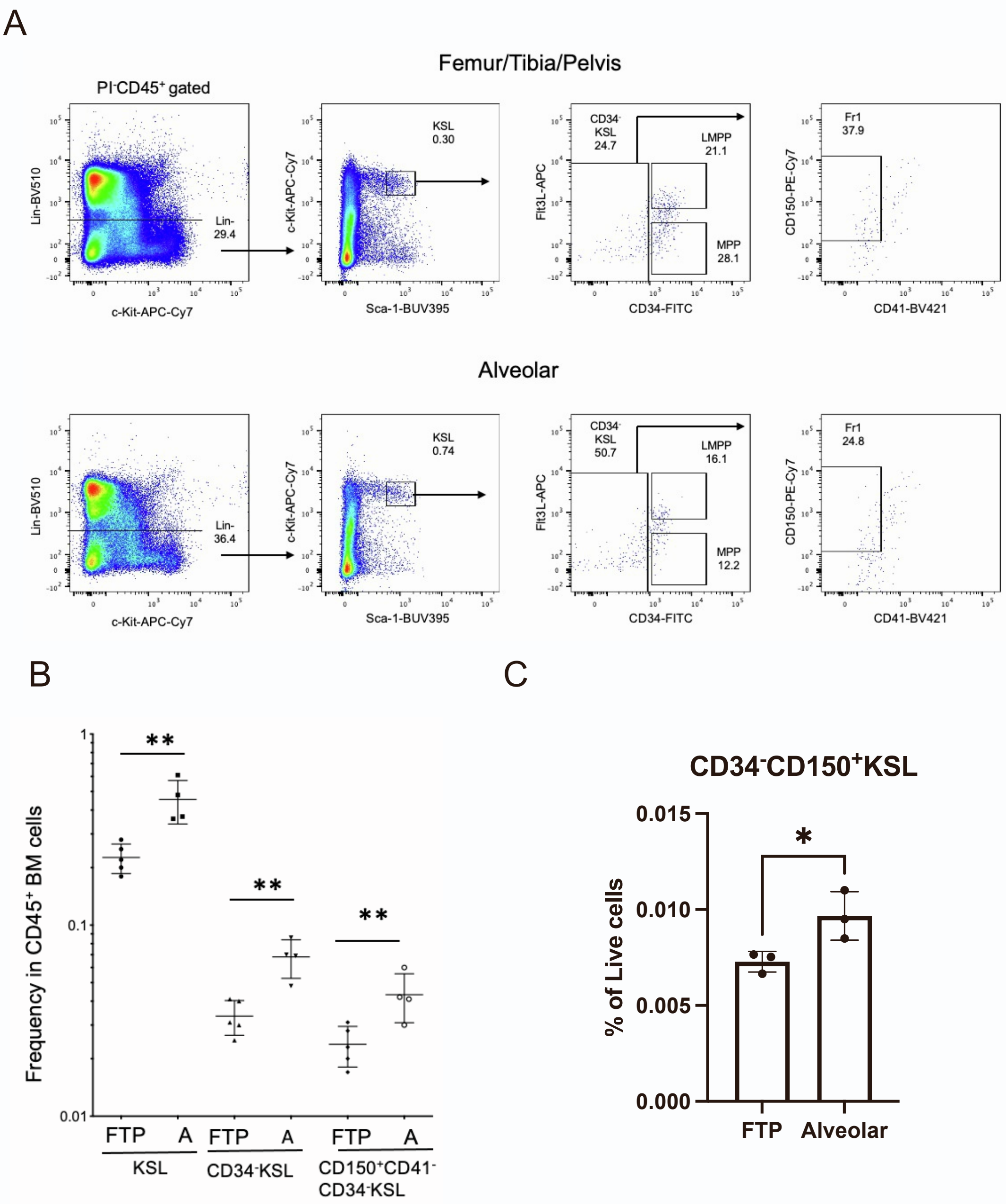

Figure S3. Frequency and Functional Analysis of HSCs in Alveolar and Femur/Tibia/Pelvis Bone Marrow.

(A) Representative flow cytometric plots showing the gating strategy for identifying KSL, $CD34^-$KSL, and $CD150^+CD41^-CD34^-$KSL cell populations in bone marrow from femur/tibia/pelvis (FTP) and alveolar regions.

(B) Quantification of KSL, $CD34^-$KSL, and $CD150^+CD41^-CD34^-$KSL cells in bone marrow from FTP and alveolar regions, expressed as a percentage of $CD45^+$ cells. Data are presented as mean ± SEM. Statistical significance was determined using an unpaired two-tailed Student's t-test: **$p < 0.01$. Data are presented as mean ± SEM from four independent experiments ($n = 4$).

(C) Frequency of $CD34^-CD150^+$KSL cells in live cells from FTP and alveolar bone marrow. Data are presented as mean ± SEM. Statistical significance was determined using an paired two-tailed Student's t-test: *$p < 0.05$. Data are presented as mean ± SEM from three independent experiments ($n = 3$).

Figure S4

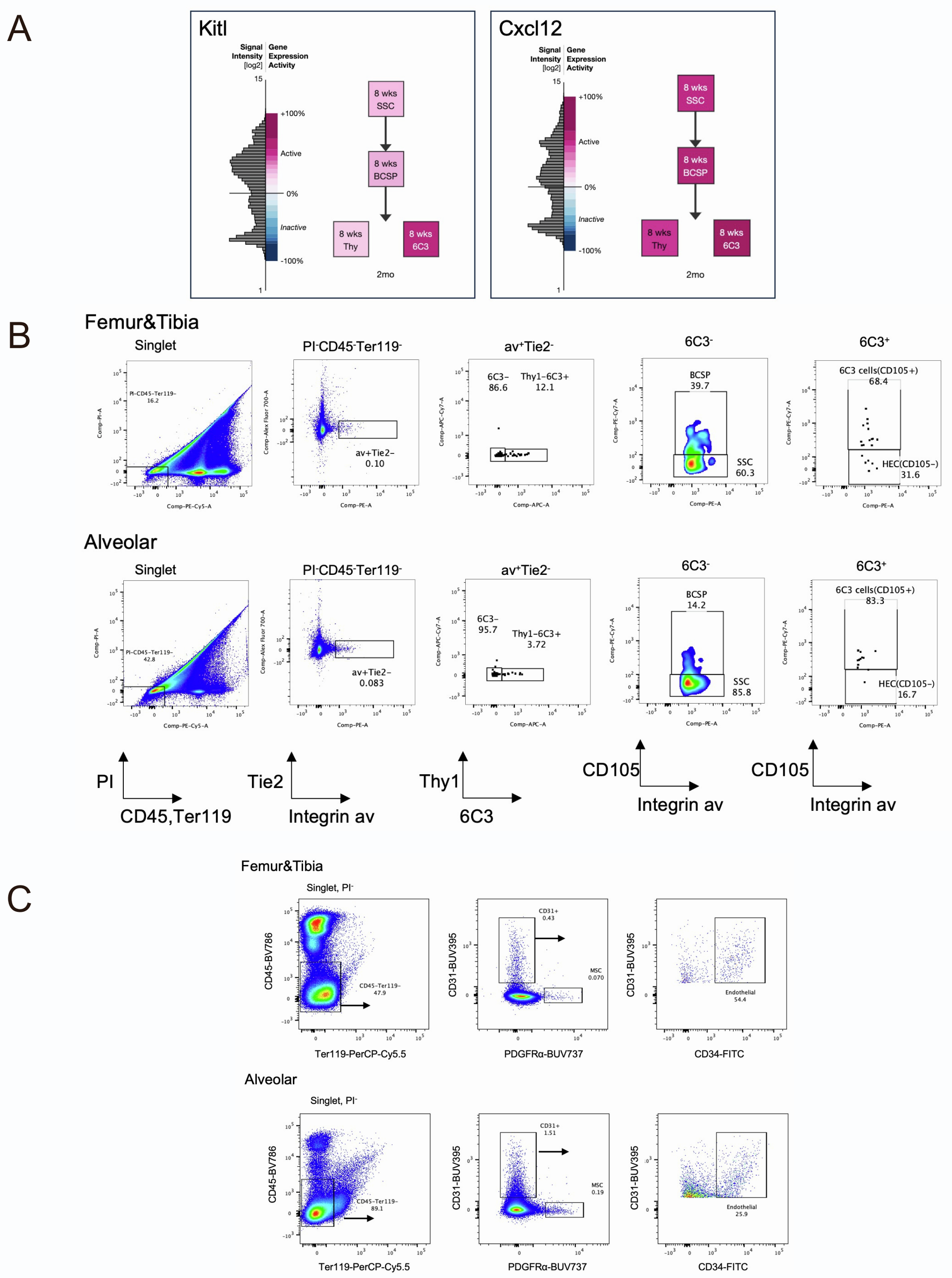

Figure S4 Expression and Identification of Skeletal, Mesenchymal, and Endothelial Cell Populations in Alveolar and Femur/Tibia Bone Marrow

(A) Expression of *Kitl* and *Cxcl12 in skeletal stem cell lineages*

Relative expression of *Kitl* and *Cxcl12* in skeletal stem cell lineages of 8-week old mouse as determined by Affymetrix Mouse Genome 430 2.0 Array analysis. Pink represents increased expression, blue represents decreased expression.

(B) Identification of Skeletal Stem and Progenitor Cell Populations in Alveolar and Femur/Tibia Bone Marrow. Representative flow cytometry plots for the identification of skeletal stem cells (SSC; CD45^{-}Ter-119^{-}Tie2^{-}AlphaV^{+}Thy^{-}6C3^{-}CD105^{-}), bone, cartilage, stromal progenitors (BCSP; CD45^{-}Ter-119^{-}Tie2^{-}AlphaV^{+}Thy^{-}6C3^{-}CD105^{+}), Thy subpopulation (CD45^{-}Ter-119^{-}Tie2^{-}AlphaV^{+}Thy^{+}6C3–CD105^{+}), and 6C3 subpopulation (CD45^{-}Ter-119^{-}AlphaV^{+}Thy^{-}6C3^{+}CD105^{+}) in ftp-BM and al-BM.

(C)Analysis of Mesenchymal Stromal and Endothelial Cells in Alveolar and Femur/Tibia Bone Marrow. Flow cytometry plots showing the identification of mesenchymal stromal cells (MSC; CD45^{-}Ter-119^{-}CD31^{-}PDGFRα^{+}), and endothelial cells (CD45^{-}Ter-119^{-}CD31^{+}CD34^{+}) in ft-BM and al-BM.
