# Supplementary material for: Elevated hematopoietic stem cell frequency in mouse alveolar bone marrow
Source: Stem Cell Reports. 2024 Dec 12;20(1):102374. doi: 10.1016/j.stemcr.2024.11.004 (PMC11784484; doi:10.1016/j.stemcr.2024.11.004)
Supplement: Document S2. Article plus supplemental information [file mmc2.pdf]

Stem Cell Reports
Report
OPEN ACCESS

Elevated hematopoietic stem cell frequency in mouse alveolar bone marrow

Kouta Niizuma,[1,2,12] Satoru Morikawa,[1,2,7,12] Eric Gars,[1] Jinyi Xiang,[1,4] Tomoko Matsubara-Takahashi,[8] Rei Saito,[8] Eri Takematsu,[1,3] Yuting Wang,[1,3] Haojun Xu,[5] Arata Wakimoto,[11] Tze Kai Tan,[1,2] Yoshiaki Kubota,[8] Charles K.F. Chan,[1,3] Irving L. Weissman,[1,4] Taneaki Nakagawa,[7] Adam C. Wilkinson,[1,2,9] Hiromitsu Nakauchi,[1,2,6,*] and Ryo Yamamoto[1,2,10,13,*]

[1]Institute for Stem Cell Biology and Regenerative Medicine, Stanford University School of Medicine, Stanford, CA 94305, USA
[2]Department of Genetics, Stanford University School of Medicine, Stanford, CA 94305, USA
[3]Department of Surgery, Division of Plastic and Reconstructive Surgery, Stanford University School of Medicine, Stanford, CA 94305, US
[4]Department of Pathology, Stanford University School of Medicine, Stanford, CA 94305, USA
[5]Department of Neurology, Stanford University School of Medicine, Stanford, CA 94305, USA
[6]Division of Stem Cell Therapy, Distinguished Professor Unit, Institute of Medical Science, The University of Tokyo, Tokyo, Japan
[7]Department of Dentistry and Oral Surgery, Keio University School of Medicine, Tokyo, Japan
[8]Department of Anatomy, Keio University School of Medicine, Tokyo, Japan
[9]MRC Weatherall Institute of Molecular Medicine, Radcliffe Department of Medicine, University of Oxford, Oxford, UK
[10]Institute for the Advanced Study of Human Biology (WPI-ASHBi), Kyoto University, Kyoto 606-8501, Japan
[11]Ph.D. Program in Human Biology, School of Integrative and Global Majors, University of Tsukuba, Ibaraki 305-8575, Japan
[12]These authors contributed equally
[13]Lead contact
*Correspondence: nakauchi@stanford.edu (H.N.), yamamoto.ryo.2c@kyoto-u.ac.jp (R.Y.)
https://doi.org/10.1016/j.stemcr.2024.11.004

SUMMARY

Hematopoietic stem cells (HSCs) are crucial for maintaining hematopoietic homeostasis and are localized within distinct bone marrow (BM) niches. While BM niches are often considered similar across different skeletal sites, we discovered that the alveolar BM (al-BM) in the mandible harbors the highest frequency of immunophenotypic HSCs in nine different skeletal sites. Transplantation assays revealed significantly increased engraftment from al-BM compared to femur, tibia, or pelvis BM, likely due to a higher proportion of alveolar HSCs. Moreover, hematopoietic progenitor cells (c-Kit$^+$ Sca-1$^+$ Lin$^-$) in al-BM exhibited increased quiescence and reduced apoptosis, indicating superior maintenance and survival characteristics. We also observed an enrichment of mesenchymal stromal cells and skeletal stem cells in al-BM, suggesting a more supportive microenvironment. These findings indicate that al-BM provides a unique microenvironment conducive to higher frequency of HSCs, offering new insights into site-specific hematopoiesis.

INTRODUCTION

Hematopoietic stem cells (HSCs) support the life-long homeostasis of the hematopoietic system, and their therapeutic application in hematopoietic stem cell transplantation provides a curative treatment for a wide range of hematological malignant and non-malignant diseases (Orkin and Zon, 2008; Eaves, 2015). Most adult HSCs reside within the complex BM microenvironment or niche, which is thought to tightly regulate HSC activity. Mounting evidence highlights the importance of precise niche regulation of HSCs for their function and long-term maintenance, with niche corruption being proposed as a major cause of hematopoietic system dysfunction (Crane et al., 2017; Szade et al., 2018; Pinho and Frenette, 2019).

Our team has recently developed two key systems: a chemically defined *ex vivo* culture system for expanding functional mouse HSCs—up to 900-fold over 28 days, and a cytokine-free, chemically defined culture system for functional human HSCs—up to 55-fold over 30 days (Wilkinson et al., 2019; Igarashi et al., 2023; Sakurai et al., 2023). These *ex vivo* HSC expansion methods open the possibility of expanding small numbers of long-term HSCs (LT-HSCs) into the quantities required for clinical hematopoetic stem cell transplantaion. However, such methods have shown that it is possible to reproduce HSC self-renewal without signals from the BM niche, but they do not lead to an understanding of the anatomical location or function of the bone marrow niche. This understanding is critical, as variability in HSC properties across different sites could impact the efficiency and effectiveness of their expansion and subsequent clinical applications. Although significant differences in hematopoiesis are known to exist within the fetal liver and spleen (Morita et al., 2011), definitive hematopoiesis in various adult mouse BM compartments has been assumed to be similar (Kiel et al., 2005). Little is known about spatial differences in hematopoiesis in adult BM; most current knowledge in mice comes from studies using BM from femur, tibia (ft-BM), sternum, and calvaria (Kunisaki et al., 2013; Acar et al., 2015; Kokkaliaris et al., 2020; Christodoulou et al., 2020).

Using multicolor flow cytometry, we characterized hematopoiesis in nine different skeletal sites and unexpectedly discovered that mouse al-BM had the highest frequency of hematopoietic stem/progenitor cells (HSPCs) among them.

© 2024 The Authors.
Published by Elsevier Inc. on behalf of International Society for Stem Cell Research.
This is an open access article under the CC BY license (http://creativecommons.org/licenses/by/4.0/).

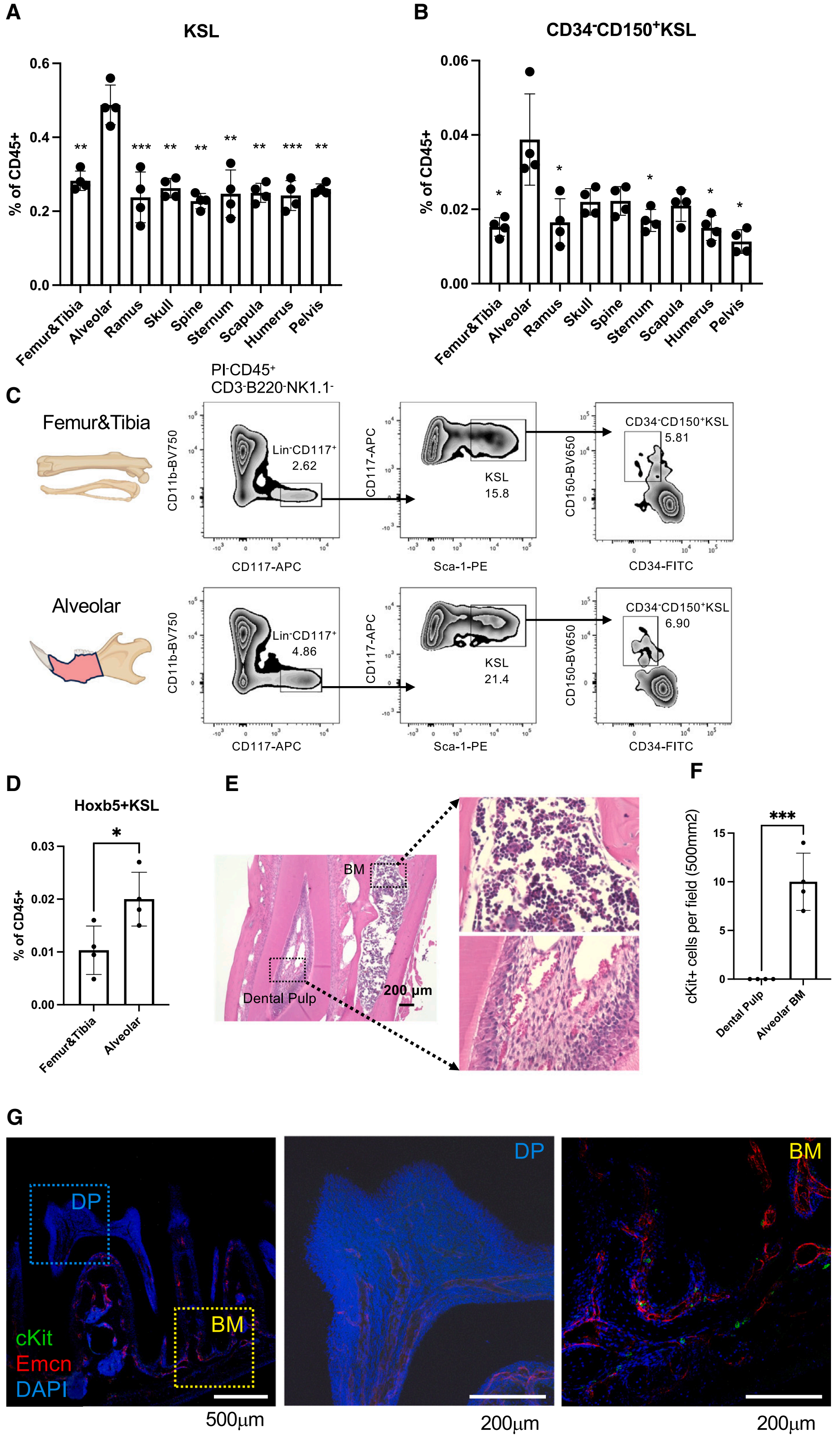

(legend on next page)

RESULTS

HSPC comparison across different bones

To explore site-specific hematopoiesis phenotypes, we isolated nine bone types (femur and tibia, alveolar, ramus, calvarium, spine, sternum, scapula, humerus, and pelvis) from adult mice. We assessed the frequency (as percentage of $CD45^+$ cells) of immunophenotypic HSPCs (KSL; $c\text{-}Kit^+Sca\text{-}1^+Lin^-$) and HSCs ($CD34^-CD150^+c\text{-}Kit^+$ $Sca\text{-}1^+Lin^-$) in these bone types. Surprisingly, al-BM showed the highest mean percentages of KSL and HSC populations, approximately twice those found in ft-BM (Figures 1A–1C and S1A). We also observed the elevated frequency of $Lin^-c\text{-}Kit^+$ cells (KL) in the al-BM (Figure S1B). Analysis of various immune cell populations, including $CD3^+$ T cells, $B220^+$ cells, Mo/Mac (monocytes/macrophages, $CD11b^+Ly6G^-$), Neu (neutrophils, $CD11b^+Ly6G^+$), and NK (natural killer, $NK1.1^+$) cells, revealed no significant differences in their frequencies across the different bone types (Figure S1D). Considering the lack of reports on HSCs in mouse al-BM, we focused on characterizing HSCs in this region (Figure S1C).

Increased frequency of LT-HSCs in al-BM identified by HOXB5 reporter

In light of the established utility of the HOXB5 marker in identifying LT-HSCs (Chen et al., 2016), we utilized the HOXB5 reporter mouse model to investigate the frequency of LT-HSCs in different BM compartments. Our flow cytometry analysis demonstrated a higher frequency of $mCherry^+KSL$ (=$HOXB5^+$) cells in al-BM compared to ft-BM, suggesting a higher concentration of LT-HSCs in this compartment (Figures 1D and S2A).

Localization of HSCs in al-BM

To further examine the presence and distribution of hematopoietic cells, we performed hematoxylin and eosin (H&E) staining on sections of the mandible bone (Figures 1E, S2B, and S2C). Our analysis revealed a notable presence of hematopoietic cells within the al-BM compartment (Figure 1E), characterized by distinct cellular morphology and organization typical of active hematopoietic tissue. In contrast, the dental pulp (DP) showed a minimal presence of such cells (Figure 1E). This stark difference underscores the unique hematopoietic activity in the al-BM compared to DP.

Moreover, to pinpoint the anatomical location of HSPCs, we conducted immunostaining on mandible sections from adult mice according to our previous study (Matsubara et al., 2022). This revealed the presence of $c\text{-}Kit^+$ cells in the al-BM (Figures 1F and 1G). Similar results were obtained in P18 mice (Figure S2D). The presence of $c\text{-}Kit^+$ cells in the DP was found to be almost nonexistent, which is consistent with a previous study that examined the prevalence of HSPCs and immune cells in the DP during steady state (Osaki et al., 2022). These observations, marked by the presence of $c\text{-}Kit^+$ cells in the al-BM, suggest potential sites of HSCs and active hematopoiesis in this region.

Higher frequency of functional HSCs in al-BM compared to femur, tibia, and pelvis BM

Given the lack of significant differences in HSC frequency among femur, tibia, and pelvis, we pooled BM cells from these sites for analysis. Flow cytometric analysis of this pooled BM (femur, tibia, and pelvis; ftp-BM) and al-BM confirmed the higher frequency (as percentage of $CD45^+$ cells and live cells) of immunophenotypic HSCs in the alveolar bone (Figures S3A–S3C). To assess the functional capacity of HSCs in al-BM, we transplanted whole al-BM and ftp-BM into lethally irradiated mice, alongside competitor whole BM cells. al-BM cells reconstituted more $CD45^+$ cells in peripheral blood (PB) than ftp-BM cells in both primary and secondary recipient mice (Figure 2A), consistent with the increased frequency of immunophenotypic HSCs.

Comparative functional analysis of al-BM and ftp-BM HSCs

To further investigate whether the reconstitution potential of al-BM HSCs exceeded that of ftp-BM, we transplanted 10 immunophenotypic ($CD34^-KSL$) HSCs from each source

Figure 1. Characterization of the immunophenotypic hematopoietic stem/progenitor cells in al-BM

(A) Frequency of KSL within the hematopoietic compartment ($CD45^+$) in al-BM compared to other bone types ($n = 4$).

(B) Frequency of HSCs within $CD45^+$ in al-BM compared to other bone types ($n = 4$).

(C) Representative flow cytometric data of al-BM and ft-BM.

(D) Frequency of Hoxb5-$mCherry^+$LT-HSCs in al-BM and ft-BM of Hoxb5 reporter mice ($n = 4$). Data are presented as mean ± SEM. Statistical significance was determined using a paired two-tailed Student's t test: $*p < 0.05$.

(E) H&E staining of mandible sections.

(F) Quantification of $c\text{-}Kit^+$ cells in al-BM and DP sections using immunohistochemistry ($n = 4$). Data are presented as mean ± SEM. Statistical significance was determined using an unpaired two-tailed Student's t test: $***p < 0.001$.

(G) Immunohistochemistry for a mandible from an adult mouse. Confocal images showing the presence of $c\text{-}Kit^+$ cells (green), $Endomucin^+$ cells (red), and DAPI-stained nuclei (blue) in al-BM. Data are presented as mean ± SEM. $*p < 0.05$, $**p < 0.01$, $***p < 0.001$: paired two-tailed Student's t test (A, B, D, and F).

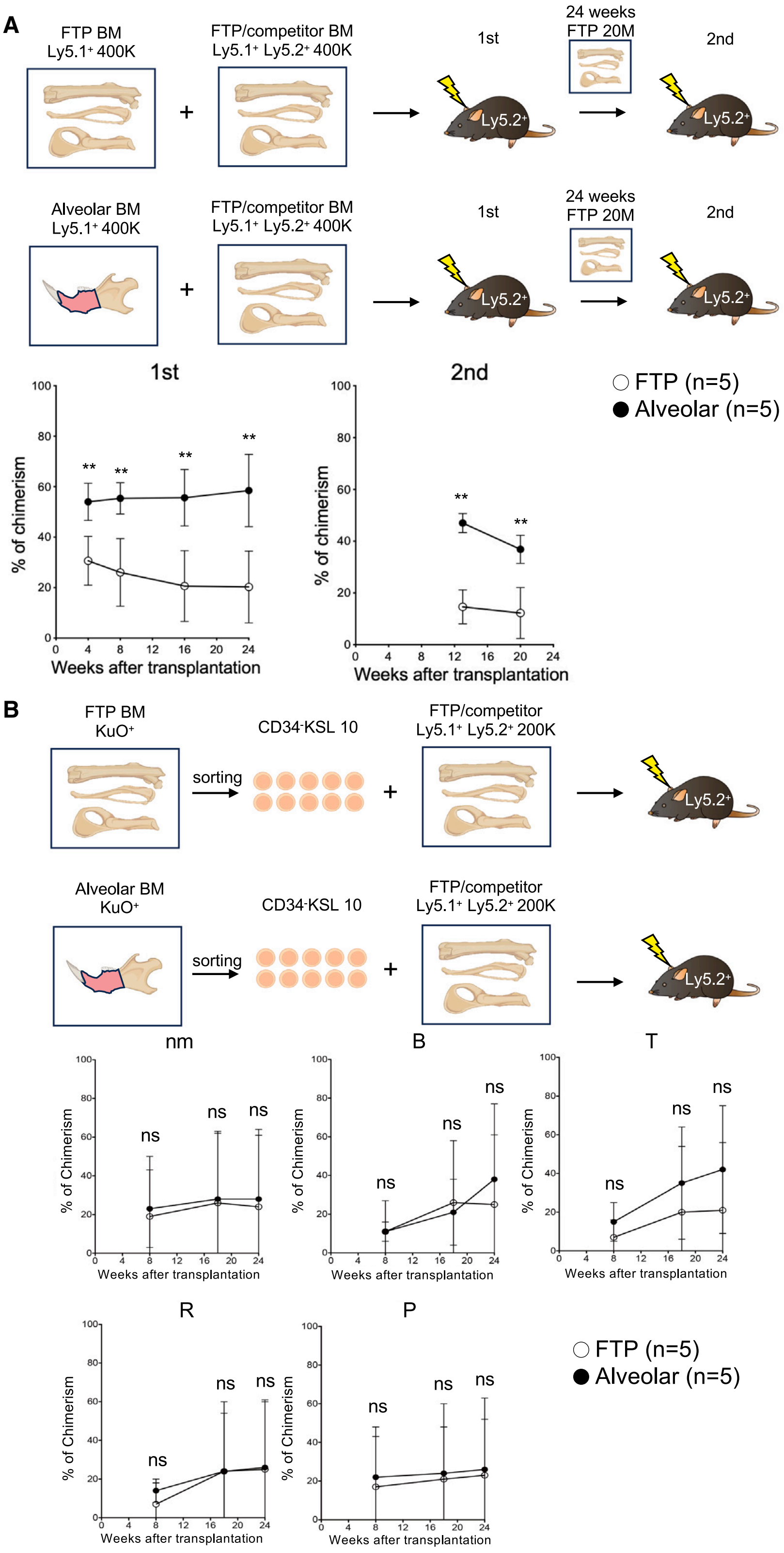

Figure 2. Comparative analysis of functional HSCs in al-BM and FTP-BM

(A) PB chimerism data from transplantation assays using whole BM ($n = 5$). * denotes $p < 0.05$, ** $p < 0.01$.

(B) Transplantation of isolated CD34⁻KSL cells from KuO mice. Ten CD34⁻KSL cells from either ftp-BM or al-BM were transplanted into lethally irradiated Ly5.2 mice with 2×10^5 competitor cells from ftp-BM of Ly5.1/Ly5.2-F1 mice. Chimerism in PB, reflecting the engraftment efficiency of transplanted cells, was analyzed at specified intervals ($n = 5$). Lineage-specific chimerism is shown for neutrophils and monocytes (nm), B cells (B), T cells (T), red blood cells (R), and platelets (P). Data are presented as mean ± SEM. Statistical significance was determined using an unpaired two-tailed Student's t test for each comparison. ns denotes non-significant differences.

into lethally irradiated mice, alongside competitor whole BM cells. The results demonstrated that HSCs from both al-BM and ftp-BM were equally effective in reconstituting the five hematopoietic lineages in PB (Figure 2B), indicating functional equivalence between HSCs in al-BM and those in ftp-BM.

Greater expandability of al-BM cells in a PVA-based HSC medium

We previously developed a PVA-containing culture medium that supports mouse HSCs *ex vivo* (Wilkinson et al., 2019), and it has recently been found to enrich HSPCs from BM cells (Ochi et al., 2021). We hypothesized that al-BM might contain a greater number of expandable cells than ft-BM in this PVA-based HSC medium. To test this hypothesis, we isolated al- and ft-BM, sorted 10K $CD45^+$ hematopoietic cells per well, and cultured them for 14 days *ex vivo* (Figure 3A). As a result, the total number of live cells was significantly higher in cultures derived from al-BM than those from ft-BM (Figure 3B). Moreover, we detected a higher percentage of KL fraction in al-BM compared to ft-BM but no significant difference in the frequency of KSL and $CD150^+$KSL (Figure 3C). These data indicate that al-BM contains more expandable cells than ft-BM in the PVA-based medium, suggesting that al-BM cells may sustain hematopoietic progenitor cells rather than immunophenotypic HSC populations in this culture system. The higher expansion of al-KL cells could be attributed to the unique microenvironment of the al-BM, which may provide distinct signaling cues and niche factors that preferentially support the proliferation and maintenance of hematopoietic progenitor cells.

Functional comparability of al-BM and ft-BM HSCs in the PVA-based HSC medium

Next, to determine whether there is a functional difference between al-BM HSCs and ft-BM HSCs in the PVA-based medium, we cultured 50 $CD34^-$KSL HSCs from al-BM and ft-BM (Figure 3D). After 14 days of culture, we analyzed the cell number and immunophenotype by flow cytometry. No significant differences were observed between al-BM and ft-BM (Figures 3E and 3F). These results suggest that HSCs in al-BM are functionally compatible with those in ft-BM in the PVA-based culture system.

Cell-cycle status and survival of KSL cells from al-BM and ft-BM

We analyzed the cell-cycle status and survival/apoptosis of KSL cells from al-BM and ft-BM to elucidate potential biological differences between these microenvironments. We detected a significantly higher percentage of KSL cells in the G0 phase in al-BM compared to ft-BM, while a higher percentage of KSL cells were observed in the S/G2/M phases in ft-BM compared to al-BM (Figure 4A). These results indicate a higher quiescent state in al-BM KSL. Furthermore, the apoptosis status analysis showed a significantly lower percentage of Annexin-V^+ KSL cells in al-BM compared to ft-BM (Figure 4B), suggesting enhanced survival in al-BM. These findings highlight distinct cell-cycle characteristics and improved survival of KSL cells in al-BM compared to ft-BM, demonstrating biological differences between these two BM microenvironments.

Higher frequencies of HSC-supporting cells in al-BM

In exploring the potential mechanisms underlying the higher frequency of HSCs and HSPCs in al-BM, our attention was drawn to the al-HSC microenvironment. Recognizing the critical role of the niche in regulating HSC/HSPC homeostasis, we examined the expression of niche factors by the gene expression commons platform (Seita et al., 2012). Our analysis, inspired by findings in skeletal stem cell (SSC) biology (Chan et al., 2015), identified that SSCs, bone, cartilage, stromal progenitors (BCSPs), Thy subpopulation ($CD45^-$Ter-119^-Tie2^-AlphaV$^+$Thy$^+$6C3^-CD105^+), and 6c3 subpopulation ($CD45^-$Ter-119^-AlphaV$^+$Thy$^-$6C3^+CD105^+) exhibit high expression levels of key HSC niche factors (*Kitl* and *Cxcl12*) (Figure S4A). This led us to hypothesize that al-BM may harbor a higher frequency of these cells compared to ft-BM. Next, we analyzed the frequency of these cell types in al-BM and ft-BM. We found that al-BM has a significantly higher frequency of SSC, BCSP, and Thy subpopulations compared to ft-BM, whereas there was no difference in the frequency of 6c3 subpopulation (Figures 4C and S4B).

Moreover, given the established importance of mesenchymal stromal cells (MSCs) and endothelial cells (ECs) in HSC maintenance (Ding et al., 2012; Ding and Morrison, 2013; Greenbaum et al., 2013; Himburg et al., 2018), we examined the frequencies of these cells in al-BM and ft-BM. We observed a significantly higher percentage of MSCs in al-BM compared to ft-BM (Figures 4D and S4C), indicating a more supportive stromal environment in al-BM. In contrast, the frequency of ECs did not differ significantly between al-BM and ft-BM (Figure 4D). These findings suggest that the enhanced HSC and HSPC frequencies in al-BM may be attributed to the increased presence of MSCs.

Potential role of oncostatin M in al-BM Mo/Mac for HSC retention

Building on the understanding of niche biology, we investigated additional factors that might contribute to the unique characteristics of the al-BM niche. A recent study performing single-cell RNA sequencing of cells in mandibular alveolar bone (Lin et al., 2021) reported that Mo/Mac in the mandibular al-BM express higher levels of oncostatin M (*Osm*) compared to those in ft-BM. Given the

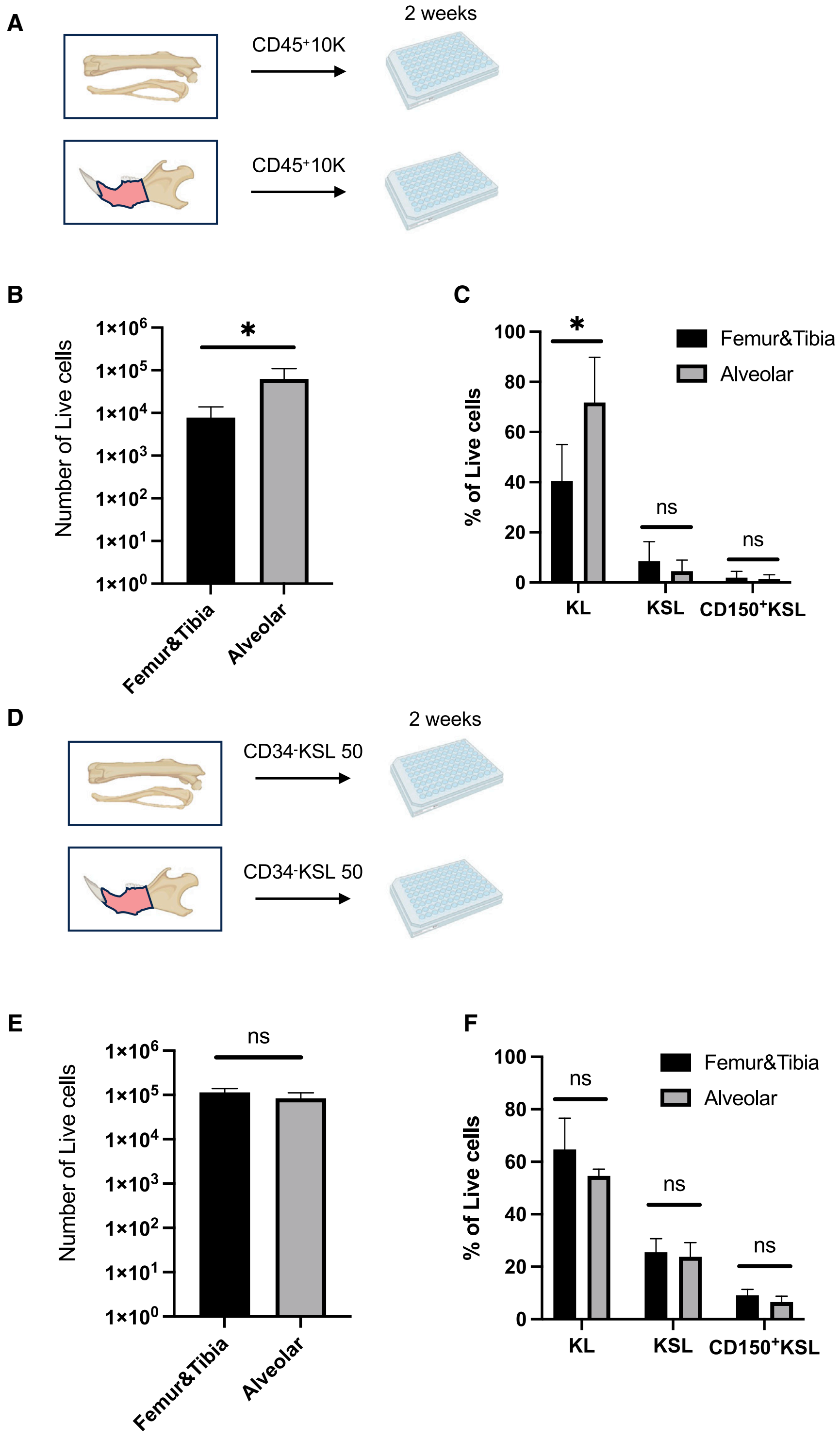

Figure 3. Comparative analysis of *ex vivo* expansion of HSCs in al-BM and FTP-BM

(A) Schematic of the experimental setup.

(B) The total number of live cells after 14 days culture ($n = 4$). Data are presented as mean ± SEM. Statistical significance was determined using an unpaired two-tailed Student's t test: $*p < 0.05$.

(C) The percentage of phenotypic KL, KSL, and $CD150^+$KSL cells derived from 10K $CD45^+$ cells from al-BM and ft-BM after 14 days of culture ($n = 4$).

(D) Schematic of the experimental setup.

(E) The total number of live cells derived from 50 $CD34^-$KSL cells cultured from al-BM and ft-BM after 14 days ($n = 4$). Data are presented as mean ± SEM. Statistical significance was determined using an unpaired two-tailed Student's t test: ns, not significant.

(F) The percentage of phenotypic KL, KSL, and $CD150^+$KSL cells derived from 50 $CD34^-$KSL cells from al-BM and ft-BM after 14 days of culture ($n = 4$). Data are presented as mean ± SEM. Statistical significance was determined using an unpaired two-tailed Student's t test: $*p < 0.05$, ns, not significant.

reported importance of OSM in restricting HSC mobilization in BM (Bisht et al., 2022), we hypothesized that this cytokine could be a key factor in the al-BM niche. Next, we isolated Mo/Mac from al-BM and ft-BM and performed quantitative PCR to assess *Osm* expression. We found that Mo/Mac from al-BM express significantly higher levels of *Osm* compared to those from ft-BM (Figure 4B), suggesting the enhanced HSC retention in al-BM via OSM.

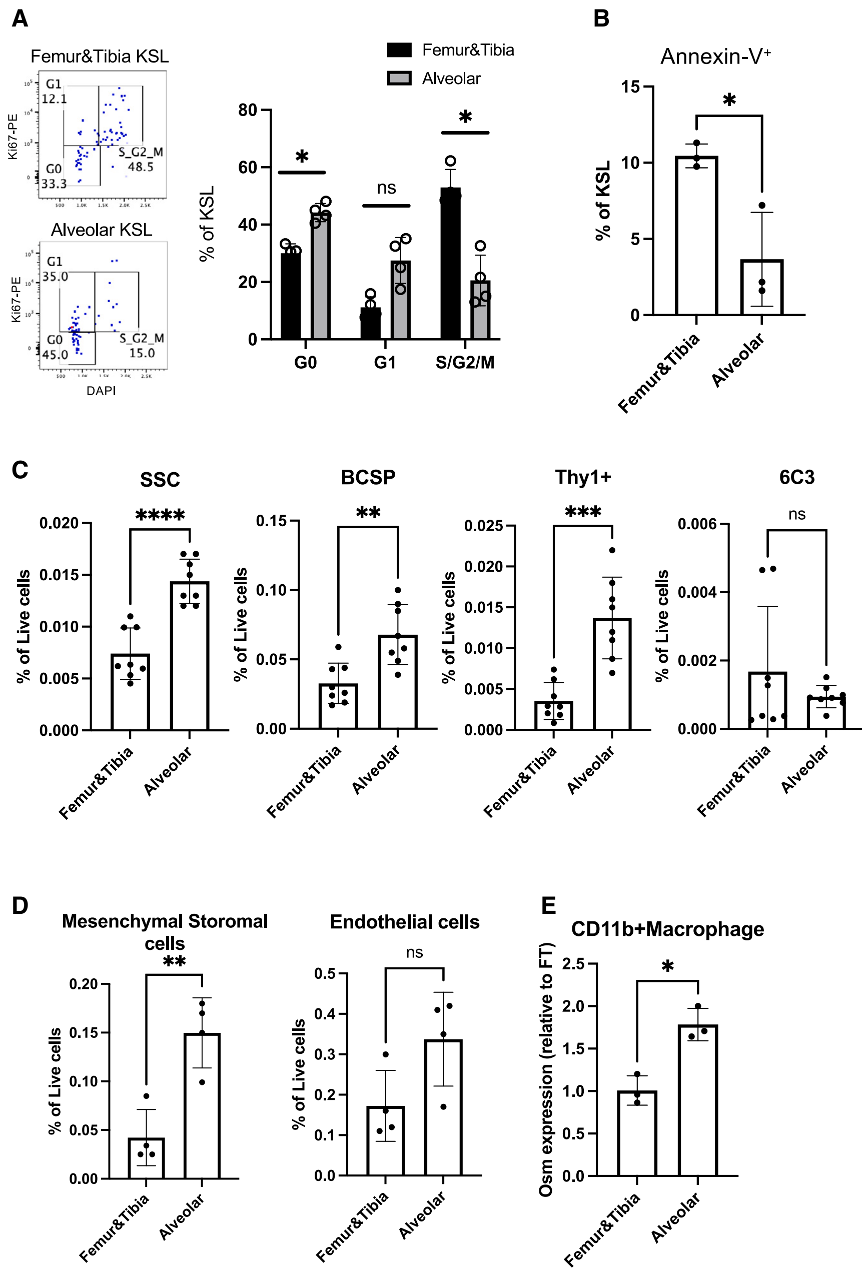

Figure 4. Analysis of microenvironment in al-BM

(A) Frequency of KSL cells in different cell-cycle phases in al-BM and ft-BM (n = 4). Statistical significance was determined using a paired two-tailed Student's t test: $*p < 0.05$.

(B) Frequency of Annexin-V$^+$ KSL cells in al-BM and ft-BM (n = 3). Statistical significance was determined using an unpaired two-tailed Student's t test: $*p < 0.05$.

(C) Frequency of niche-associated cell populations in al-BM and ft-BM (n = 8). The chart shows the percentage of skeletal stem cells (SSCs), bone, cartilage, stromal progenitors (BCSPs), Thy subpopulation, and 6C3 subpopulation as a proportion of total live cells.

(D) Frequency of MSCs and endothelial cells in al-BM and ft-BM (n = 4). Statistical significance was determined using a paired two-tailed Student's t test: $**p < 0.01$, ns, not significant.

(E) Quantitative PCR analysis of *Osm* expression in Mo/Mac (CD45$^+$CD11b$^+$Ly6g$^-$) isolated from al-BM and ft-BM (n = 3). The graph depicts the relative expression levels of *Osm*.

DISCUSSION

Our findings challenge the prevailing assumption that adult HSCs do not acquire permanent regional differences in characteristics. Previous studies by Kiel et al. found no differences among HSCs from various adult BM compartments in mice (Kiel et al., 2005). However, our extensive analysis, including nine types of bone such as the alveolar bone and mandibular ramus, reveals that the al-BM harbors a notably increased number of functional HSCs. This marks the first identification of a BM compartment in a steady state that enriches functional HSCs, broadening our understanding of HSC biology. The precise mechanisms and physiological significance behind the elevated frequency of HSCs in mouse al-BM remain elusive. We propose the following hypotheses as possible explanations for the mechanisms.

Cellular composition of the HSC microenvironment in al-BM

The qualitative and quantitative differences in the cells constituting the HSC niche in al-BM might contribute to this phenomenon. Previous studies have indicated that

MSCs from human al-BM exhibit superior osteodifferentiation potential compared to those from iliac BM (Matsubara et al., 2005), suggesting a unique site-specific phenotype of al-BM. These distinct properties of al-BM MSCs could influence the phenotype of al-HSC niche. Additionally, the embryological origin of alveolar bone, derived from the neural crest (ectoderm), differs from most bones, which are mesodermal in origin (Watson et al., 2018). This difference in cellular origin may impact the microenvironment. In this study, we found an elevated presence of HSC-supporting cells in al-BM, such as MSCs and SSCs. Further studies employing lineage tracing and single-cell RNA sequencing technologies could provide deeper insights into these microenvironmental differences.

Physical stress on alveolar bone

The alveolar bone undergoes constant mechanical stress due to mastication, leading to active bone metabolism and remodeling (Gruber, 2019). Such stimuli could alter the expression of niche factors, affecting HSC frequency.

Influence of oral microbiota and inflammation

al-BM's routine exposure to oral microbiota and associated inflammatory challenges (Irie et al., 2014) might modify the environment, potentially impacting hematopoiesis.

Oncostatin M expression in al-BM Mo/Mac

Our findings revealed higher levels of *Osm* expression in Mo/Mac from al-BM compared to ft-BM. OSM is known to play a role in HSC retention by restricting HSC mobilization. This elevated *Osm* expression may contribute to the increased HSC frequency observed in al-BM. Further *in vivo* experiments, including the use of *Osm* conditional knockout mice and *in vivo* HSC mobilization assays, are necessary to conclusively determine the role of OSM in HSC retention and frequency in al-BM.

Temperature variation

Being the only bone exposed to the external environment, the alveolar bone might experience lower temperature conditions compared to other BM sites, possibly influencing HSC frequency.

Cell cycle and survival characteristics

Our findings suggest that al-BM provides a more quiescent and supportive environment for HSCs. This might contribute to the increased frequency of HSCs in al-BM by promoting their maintenance and survival. Further studies are needed to examine whether these characteristics are consistent in the LT-HSC fraction, as our current data are limited to the KSL fraction.

While our study provides valuable insights into mouse al-BM, no existing research on hematopoiesis in human al-BM was performed. However, given the minimally invasive nature of al-BM collection in humans (Mason et al., 2014), al-BM sourced during implant surgery and jaw deformity surgery could be a valuable resource for future studies. The discovery of an HSC-enriched BM compartment in mice opens avenues for comparative studies, which could lead to the identification of novel molecular mechanisms necessary for HSC proliferation and maintenance. Understanding these mechanisms may not only provide insights into HSC biology but also inform the development of improved protocols for the *ex vivo* expansion of HSCs.

Limitations of the study

The trabecular structure of the alveolar bone made it difficult to isolate pure BM populations. Mechanical crushing led to contamination with non-hematopoietic cells, preventing us from determining absolute HSC numbers or performing experiments on highly purified subsets. In this study, we did not find any specific cell populations that were decreased in al-BM. Further analysis of progenitor populations could provide deeper insights into the differences across bone marrow sites.

EXPERIMENTAL PROCEDURES

All animal experiments were approved by the Institutional Animal Care and Use Committee of Stanford University (Protocol #33113 and #33171).

HSPC analysis

Bone marrow cells were stained with antibodies (fluorescein isothiocyanate [FITC]-CD34, PE-Sca-1, APC-c-Kit, BUV563-NK1.1, BUV-737-CD3, BV605-B220, BV650-CD150, BV750-CD11b, and BV786-CD45). Antibody staining was performed for 90 min. Following a wash step, flow cytometric analysis was performed using an FACSymphony (BD Biosciences) using propidium iodide (PI) as a dead stain. Collected data were analyzed with the FlowJo software (Tree Star, Ashland, OR).

HSC sorting and transplantation

BM cells were isolated from femur, tibia, pelvis, and alveolar bone of male young (8–12 weeks) KuO mice. These cells were then stained for 30 min with a Lin cocktail (biotinylated-CD4, -CD8, -B220/CD45RA, -TER-119, -Gr-1, and -CD127). Finally, cells were stained for 90 min with FITC-CD34, APC-c-Kit, Brilliant Violet 421-CD150, FITC-CD41, PE-Cy7-Sca-1, and streptavidin-APC/Cy7 or APC/eFluor 780 and were sorted into a 96-well plate with PBS containing 4% fetal bovine serum on the FACSAria II cell sorter (special order system) using PI as a dead stain. Competitor whole BM cells were isolated from ft-BM of male Ly5.1/Ly5.2-F1 mice, and 2×10^5 nucleated cells were transferred into 96-well plate wells. 10 HSCs (CD34-KSL) from KuO mice and competitor cells were transplanted together into lethally irradiated Ly5.2 mice (4.9 Gy x 2).

Ex vivo HSC culture

For the $CD45^+$ BM cells culture, al-BM and ft-BM were stained with Brilliant Violet 421-CD45, and $CD45^+$ cells were sorted with the FACSAria II cell sorter (BD Biosciences) using PI as a dead stain. For the HSC culture, al-BM and ft-BM were stained for 30 min with a Lin cocktail (biotinylated-CD4, -CD8, -B220/CD45RA, -TER-119, -Gr-1, and -CD127). Finally, cells were stained for 90 min with FITC-CD34, APC-cKit, Brilliant Violet 421-CD150, PE-Cy7-Sca-1, and streptavidin-APC/eFluor 780, and CD34-KSL HSCs were sorted into a 96-well plate on the SH800 cell sorter (Sony) using PI as a dead stain. 10K $CD45^+$ cells or 50 $CD34^-$KSL HSCs were cultured in media composed of F12 media (Life Technologies), 1% insulin-transferrin-selenium-ethanolamine (ITSX; Life Technologies), 1% penicillin/streptomycin/glutamine (P/S/G; Life Technologies), 10 mM HEPES (Life Technologies), 0.1% polyvinyl alcohol (Sigma, P8136), 100 ng/mL thrombopoietin, and 10 ng/mL stem cell factor on a CellBIND 96-well clear flat bottom polystyrene microplate (Corning) (Wilkinson et al., 2019; Ochi et al., 2021). Cells were incubated at 37°C, and the medium was changed every other day.

RESOURCE AVAILABILITY

Lead contact

Further information and requests for resources and reagents should be directed to and will be fulfilled by the lead contact, Ryo Yamamoto (yamamoto.ryo.2c@kyoto-u.ac.jp).

Materials availability

This study did not generate new unique reagents.

Data and code availability

- This study did not generate datasets deposited in public repositories.
- This paper does not report original code.
- Any additional information required to reanalyze the data reported in this paper is available from the lead contact upon request.

ACKNOWLEDGMENTS

We thank the Stanford Stem Cell Institute FACS Core for access to flow cytometry facilities, Catherine Carswell-Crumpton and Cheng Pan for their advice on flow cytometric assays, Kyomi Jane Igarashi for her advice on *ex vivo* HSC culture, Satoru Matsunaga for his advice on mandibular histology analysis, and Shintaro Kinoshita and Alyssa Hirakata Chang for their experimental assistance. We also acknowledge the National Institutes of Health (NIH) for supporting our work through grants R01DK116944 and R01HL147124. R.Y. is supported by Suntory Rising Stars Encouragement Program in Life Sciences (SunRiSE).

AUTHOR CONTRIBUTIONS

Conceptualization, K.N., S.M., H.N., and R.Y.; methodology, K.N., S.M., T.M.-T., Y.K., A.C.W., C.K.F.C., I.L.W., and R.Y.; validation, K.N., S.M., E.G., T.M.-T., Y.K., J.X., E.T., Y.W., H.X., R.S., T.K.T., and R.Y.; formal analysis, K.N., S.M., T.M.-T., Y.K., and R.Y.; investigation, K.N., S.M., A.W., T.M.-T., Y.K., and R.Y.; resources, R.Y., T.N., and H.N.; data curation, K.N., S.M., T.M.-T., Y.K., and R.Y.; writing – original draft preparation, K.N. and S.M.; writing – review and editing, R.Y., H.N., A.C.W., and T.N.; visualization, K.N., S.M., and R.Y.; supervision, R.Y. and H.N.; project administration, R.Y. and H.N.; funding acquisition, T.N., R.Y., and H.N. All authors have read and agreed to the published version of the manuscript.

DECLARATION OF INTERESTS

H.N. is a co-founder, member of the scientific advisory board, and shareholder of Megakaryon Corp., and Century Therapeutics, Inc.

SUPPLEMENTAL INFORMATION

Supplemental information can be found online at https://doi.org/10.1016/j.stemcr.2024.11.004.

Received: September 16, 2024
Revised: November 11, 2024
Accepted: November 12, 2024
Published: December 12, 2024

REFERENCES

Acar, M., Kocherlakota, K.S., Murphy, M.M., Peyer, J.G., Oguro, H., Inra, C.N., Jaiyeola, C., Zhao, Z., Luby-Phelps, K., and Morrison, S.J. (2015). Deep imaging of bone marrow shows non-dividing stem cells are mainly perisinusoidal. Nature *526*, 126–130.

Bisht, K., McGirr, C., Lee, S.Y., Tseng, H.W., Fleming, W., Alexander, K.A., Matsumoto, T., Barbier, V., Sims, N.A., Müller-Newen, G., et al. (2022). Oncostatin M regulates hematopoietic stem cell (HSC) niches in the bone marrow to restrict HSC mobilization. Leukemia *36*, 333–347.

Chan, C.K.F., Seo, E.Y., Chen, J.Y., Lo, D., McArdle, A., Sinha, R., Tevlin, R., Seita, J., Vincent-Tompkins, J., Wearda, T., et al. (2015). Identification and specification of the mouse skeletal stem cell. Cell *160*, 285–298.

Chen, J.Y., Miyanishi, M., Wang, S.K., Yamazaki, S., Sinha, R., Kao, K.S., Seita, J., Sahoo, D., Nakauchi, H., and Weissman, I.L. (2016). Hoxb5 marks long-term haematopoietic stem cells and reveals a homogenous perivascular niche. Nature *530*, 223–227.

Christodoulou, C., Spencer, J.A., Yeh, S.C.A., Turcotte, R., Kokkaliaris, K.D., Panero, R., Ramos, A., Guo, G., Seyedhassantehrani, N., Esipova, T.V., et al. (2020). Live-animal imaging of native haematopoietic stem and progenitor cells. Nature *578*, 278–283.

Crane, G.M., Jeffery, E., and Morrison, S.J. (2017). Adult haematopoietic stem cell niches. Nat. Rev. Immunol. *17*, 573–590.

Ding, L., and Morrison, S.J. (2013). Haematopoietic stem cells and early lymphoid progenitors occupy distinct bone marrow niches. Nature *495*, 231–235.

Ding, L., Saunders, T.L., Enikolopov, G., and Morrison, S.J. (2012). Endothelial and perivascular cells maintain haematopoietic stem cells. Nature *481*, 457–462.

Eaves, C.J. (2015). Hematopoietic stem cells: concepts, definitions, and the new reality. Blood *125*, 2605–2613.

Greenbaum, A., Hsu, Y.M.S., Day, R.B., Schuettpelz, L.G., Christopher, M.J., Borgerding, J.N., Nagasawa, T., and Link, D.C. (2013). CXCL12 in early mesenchymal progenitors is required for haematopoietic stem-cell maintenance. Nature *495*, 227–230.

Gruber, R. (2019). Osteoimmunology: Inflammatory osteolysis and regeneration of the alveolar bone. J. Clin. Periodontol. *46*, 52–69.

Himburg, H.A., Termini, C.M., Schlussel, L., Kan, J., Li, M., Zhao, L., Fang, T., Sasine, J.P., Chang, V.Y., and Chute, J.P. (2018). Distinct Bone Marrow Sources of Pleiotrophin Control Hematopoietic Stem Cell Maintenance and Regeneration. Cell Stem Cell *23*, 370–381.e5.

Igarashi, K.J., Kucinski, I., Chan, Y.Y., Tan, T.K., Khoo, H.M., Kealy, D., Bhadury, J., Hsu, I., Ho, P.Y., Niizuma, K., et al. (2023). Physioxia improves the selectivity of hematopoietic stem cell expansion cultures. Blood Adv. *7*, 3366–3377.

Irie, K., Novince, C.M., and Darveau, R.P. (2014). Impact of the Oral Commensal Flora on Alveolar Bone Homeostasis. J. Dent. Res. *93*, 801–806.

Kiel, M.J., Iwashita, T., Yilmaz, O.H., and Morrison, S.J. (2005). Spatial differences in hematopoiesis but not in stem cells indicate a lack of regional patterning in definitive hematopoietic stem cells. Dev. Biol. *283*, 29–39.

Kokkaliaris, K.D., Kunz, L., Cabezas-Wallscheid, N., Christodoulou, C., Renders, S., Camargo, F., Trumpp, A., Scadden, D.T., and Schroeder, T. (2020). Adult blood stem cell localization reflects the abundance of reported bone marrow niche cell types and their combinations. Blood *136*, 2296–2307.

Kunisaki, Y., Bruns, I., Scheiermann, C., Ahmed, J., Pinho, S., Zhang, D., Mizoguchi, T., Wei, Q., Lucas, D., Ito, K., et al. (2013). Arteriolar niches maintain haematopoietic stem cell quiescence. Nature *502*, 637–643.

Lin, W., Li, Q., Zhang, D., Zhang, X., Qi, X., Wang, Q., Chen, Y., Liu, C., Li, H., Zhang, S., et al. (2021). Mapping the immune microenvironment for mandibular alveolar bone homeostasis at single-cell resolution. Bone Res. *9*, 17.

Mason, S., Tarle, S.A., Osibin, W., Kinfu, Y., and Kaigler, D. (2014). Standardization and safety of alveolar bone-derived stem cell isolation. J. Dent. Res. *93*, 55–61.

Matsubara, T., Iga, T., Sugiura, Y., Kusumoto, D., Sanosaka, T., Tai-Nagara, I., Takeda, N., Fong, G.H., Ito, K., Ema, M., et al. (2022). Coupling of angiogenesis and odontogenesis orchestrates tooth mineralization in mice. J. Exp. Med. *219*, e20211789.

Matsubara, T., Suardita, K., Ishii, M., Sugiyama, M., Igarashi, A., Oda, R., Nishimura, M., Saito, M., Nakagawa, K., Yamanaka, K., et al. (2005). Alveolar bone marrow as a cell source for regenerative medicine: differences between alveolar and iliac bone marrow stromal cells. J. Bone Miner. Res. *20*, 399–409.

Morita, Y., Iseki, A., Okamura, S., Suzuki, S., Nakauchi, H., and Ema, H. (2011). Functional characterization of hematopoietic stem cells in the spleen. Exp. Hematol. *39*, 351–359.e3.

Ochi, K., Morita, M., Wilkinson, A.C., Iwama, A., and Yamazaki, S. (2021). Non-conditioned bone marrow chimeric mouse generation using culture-based enrichment of hematopoietic stem and progenitor cells. Nat. Commun. *12*, 3568.

Orkin, S.H., and Zon, L.I. (2008). Hematopoiesis: an evolving paradigm for stem cell biology. Cell *132*, 631–644.

Osaki, J., Yamazaki, S., Hikita, A., and Hoshi, K. (2022). Hematopoietic progenitor cells specifically induce a unique immune response in dental pulp under conditions of systemic inflammation. Heliyon *8*, e08904.

Pinho, S., and Frenette, P.S. (2019). Haematopoietic stem cell activity and interactions with the niche. Nat. Rev. Mol. Cell Biol. *20*, 303–320.

Sakurai, M., Ishitsuka, K., Ito, R., Wilkinson, A.C., Kimura, T., Mizutani, E., Nishikii, H., Sudo, K., Becker, H.J., Takemoto, H., et al. (2023). Chemically defined cytokine-free expansion of human haematopoietic stem cells. Nature *615*, 127–133.

Seita, J., Sahoo, D., Rossi, D.J., Bhattacharya, D., Serwold, T., Inlay, M.A., Ehrlich, L.I.R., Fathman, J.W., Dill, D.L., and Weissman, I.L. (2012). Gene Expression Commons: an open platform for absolute gene expression profiling. PLoS One *7*, e40321.

Szade, K., Gulati, G.S., Chan, C.K.F., Kao, K.S., Miyanishi, M., Marjon, K.D., Sinha, R., George, B.M., Chen, J.Y., and Weissman, I.L. (2018). Where Hematopoietic Stem Cells Live: The Bone Marrow Niche. Antioxid. Redox Signal. *29*, 191–204.

Watson, P.J., Fitton, L.C., Meloro, C., Fagan, M.J., and Gröning, F. (2018). Mechanical adaptation of trabecular bone morphology in the mammalian mandible. Sci. Rep. *8*, 7277.

Wilkinson, A.C., Ishida, R., Kikuchi, M., Sudo, K., Morita, M., Crisostomo, R.V., Yamamoto, R., Loh, K.M., Nakamura, Y., Watanabe, M., et al. (2019). Long-term ex vivo haematopoietic-stem-cell expansion allows nonconditioned transplantation. Nature *571*, 117–121.

Stem Cell Reports, Volume *20*

Supplemental Information

Elevated hematopoietic stem cell frequency in mouse alveolar bone marrow

Kouta Niizuma, Satoru Morikawa, Eric Gars, Jinyi Xiang, Tomoko Matsubara-Takahashi, Rei Saito, Eri Takematsu, Yuting Wang, Haojun Xu, Arata Wakimoto, Tze Kai Tan, Yoshiaki Kubota, Charles K.F. Chan, Irving L. Weissman, Taneaki Nakagawa, Adam C. Wilkinson, Hiromitsu Nakauchi, and Ryo Yamamoto

Figure S1

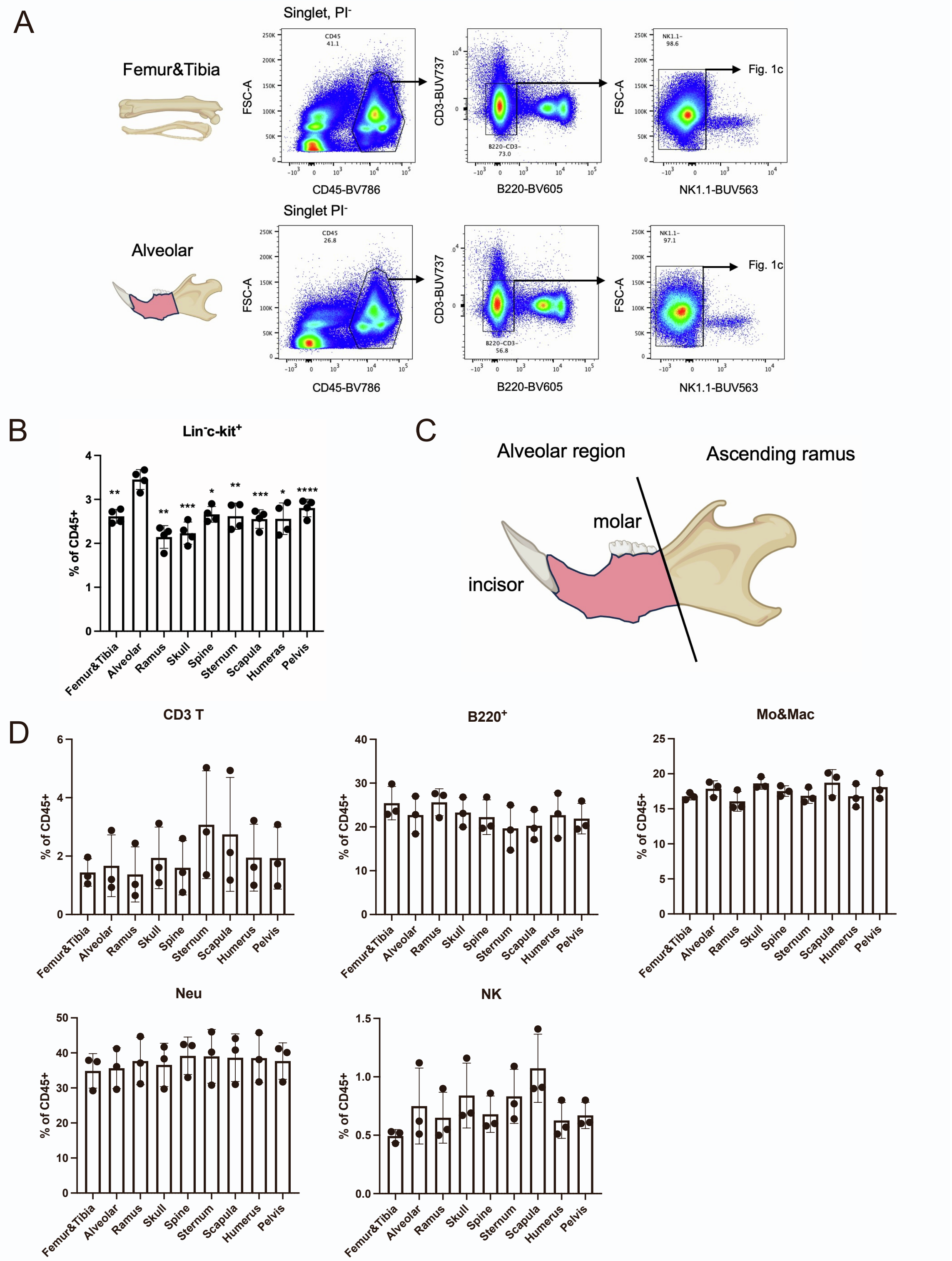

Figure S1. Enhanced Frequency of Functional HSCs in Alveolar Bone Marrow Compared to Combined Femur, Tibia, and Pelvis Bone Marrow.

(A) Representative flow cytometry plots displaying the immunophenotypic analysis of HSCs in ftp-BM (combined femur, tibia, and pelvis bone marrow) and al-BM (alveolar bone marrow) for gating out Lin^+ cells.

(B) Frequency of phenotypic hematopoietic stem/progenitor cell populations ($Lin^-c\text{-}Kit^+$) within $CD45^+$ BM cells from nine different skeletal sites, highlighting the elevated frequency in al-BM. Data are presented as mean ± SEM. Statistical significance was determined using a paired two-tailed Student's t-test for each comparison: *$p < 0.05$, **$p < 0.01$, ***$p < 0.001$, ****$p < 0.0001$. Data are presented as mean ± SEM from four independent experiments (n = 4).

(C) Schematic illustration of the mandibular bone regions, including the alveolar region and ascending ramus, used for the isolation of al-BM.

(D) The graphs display the frequency of different immune cell populations within the $CD45^+$ cell compartment across nine different skeletal sites. The immune cell populations analyzed include: $CD3^+$ T cells, $B220^+$ cells, Mo/Mac (monocytes/macrophages, $CD11b^+Ly6G^-$), Neu (neutrophils, $CD11b^+Ly6G^+$), and NK (natural killer, $NK1.1^+$). The data are presented as mean ± SEM, and no statistically significant differences were observed in the frequencies of these immune cell populations among the different bone marrow sites. Data are presented as mean ± SEM from three independent experiments (n = 3).

Figure S2

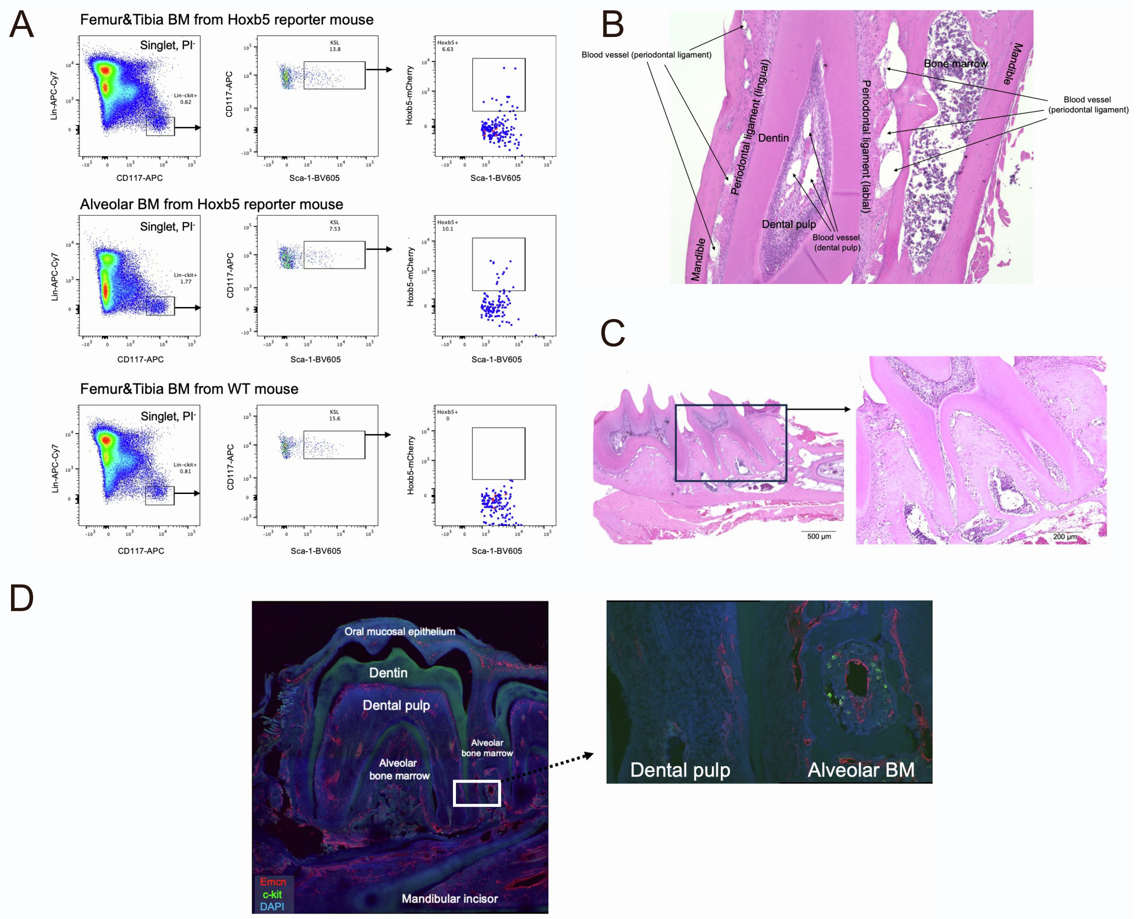

Figure S2 Identification and Histological Analysis of Hematopoietic Stem/Progenitor Cells in Alveolar and Femur/Tibia Bone Marrow

(A) Identification of Long-term Hematopoietic Stem Cells Using Hoxb5 Reporter Mouse. Representative flow cytometry plots displaying the gating strategy for identifying KSL and Hoxb5^{-}mCherry^{+} KSL populations. Data are shown for femur/tibia bone marrow (ft-BM) and alveolar bone marrow (al-BM) from Hoxb5 reporter mice, as well as femur/tibia bone marrow (ft-BM) from wild-type (WT) mice as a control.

(B) Representative H&E-stained section of the mandible, showing various structures including the dentin, periodontal ligament, dental pulp, and bone marrow. The periodontal ligament and dental pulp contain blood vessels, while the bone marrow shows distinct hematopoietic cell populations.

(C) Overview of the mandible section with detailed inset. The left image shows a low magnification view of the mandible section, highlighting the regions of interest. The right image is a higher magnification view of the boxed area, detailing the alveolar bone marrow and adjacent structures. Scale bars: 500 μm (left) and 200 μm (right).

(D) Immunohistochemical Analysis of Alveolar Bone Marrow and Dental Pulp. Composite image of a mandibular section from a P18 mouse stained for Emcn (red), c-Kit (green), and DAPI (blue). The left panel shows a low magnification view of the mandibular incisor region, while the right panel shows a higher magnification view of the alveolar BM and dental pulp.

Figure S3

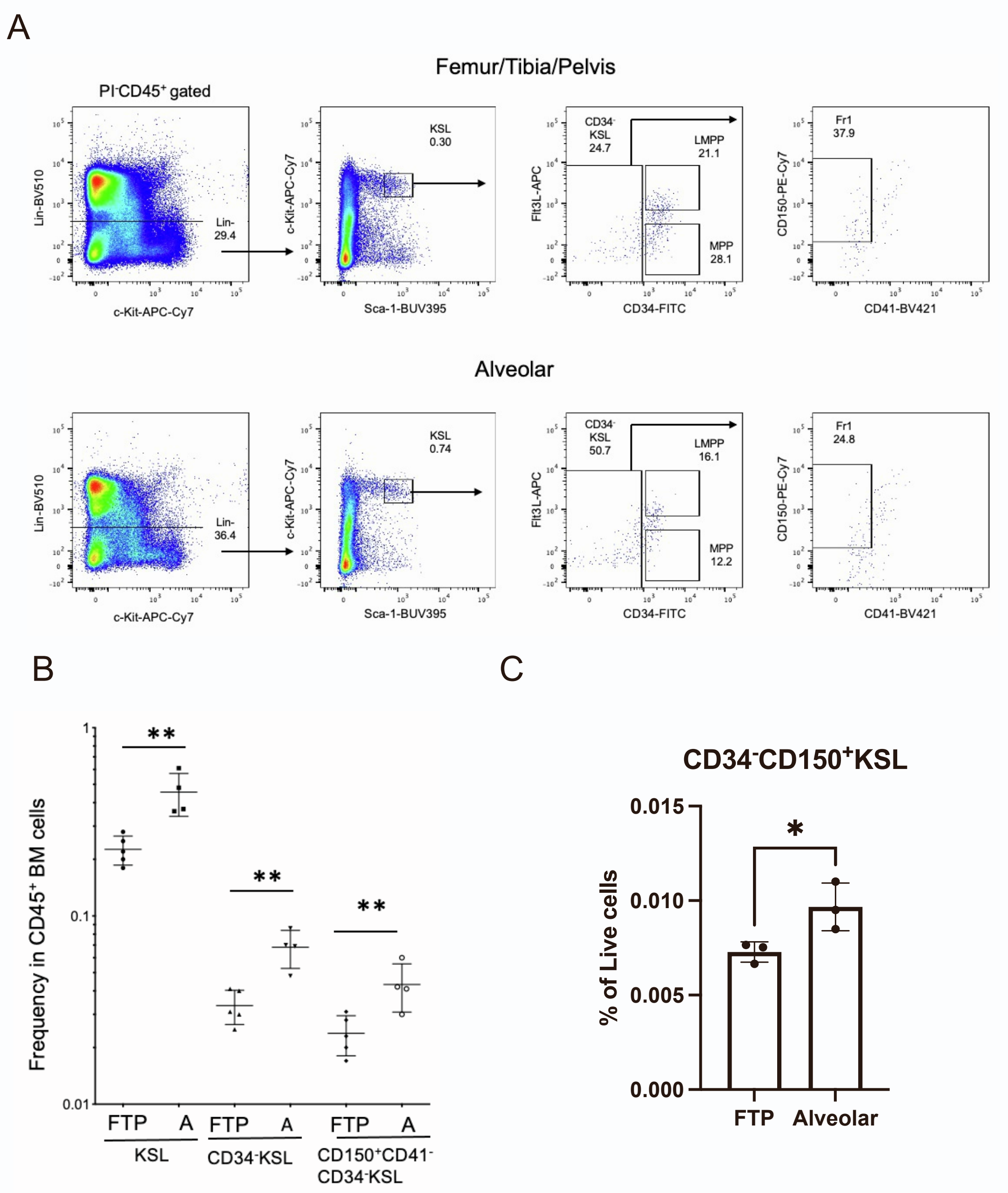

Figure S3. Frequency and Functional Analysis of HSCs in Alveolar and Femur/Tibia/Pelvis Bone Marrow.

(A) Representative flow cytometric plots showing the gating strategy for identifying KSL, CD34$^-$KSL, and CD150$^+$CD41$^-$CD34$^-$KSL cell populations in bone marrow from femur/tibia/pelvis (FTP) and alveolar regions.

(B) Quantification of KSL, CD34$^-$KSL, and CD150$^+$CD41$^-$CD34$^-$KSL cells in bone marrow from FTP and alveolar regions, expressed as a percentage of CD45$^+$ cells. Data are presented as mean ± SEM. Statistical significance was determined using an unpaired two-tailed Student's t-test: **$p < 0.01$. Data are presented as mean ± SEM from four independent experiments ($n = 4$).

(C) Frequency of CD34$^-$CD150$^+$KSL cells in live cells from FTP and alveolar bone marrow. Data are presented as mean ± SEM. Statistical significance was determined using an paired two-tailed Student's t-test: *$p < 0.05$. Data are presented as mean ± SEM from three independent experiments ($n = 3$).

Figure S4

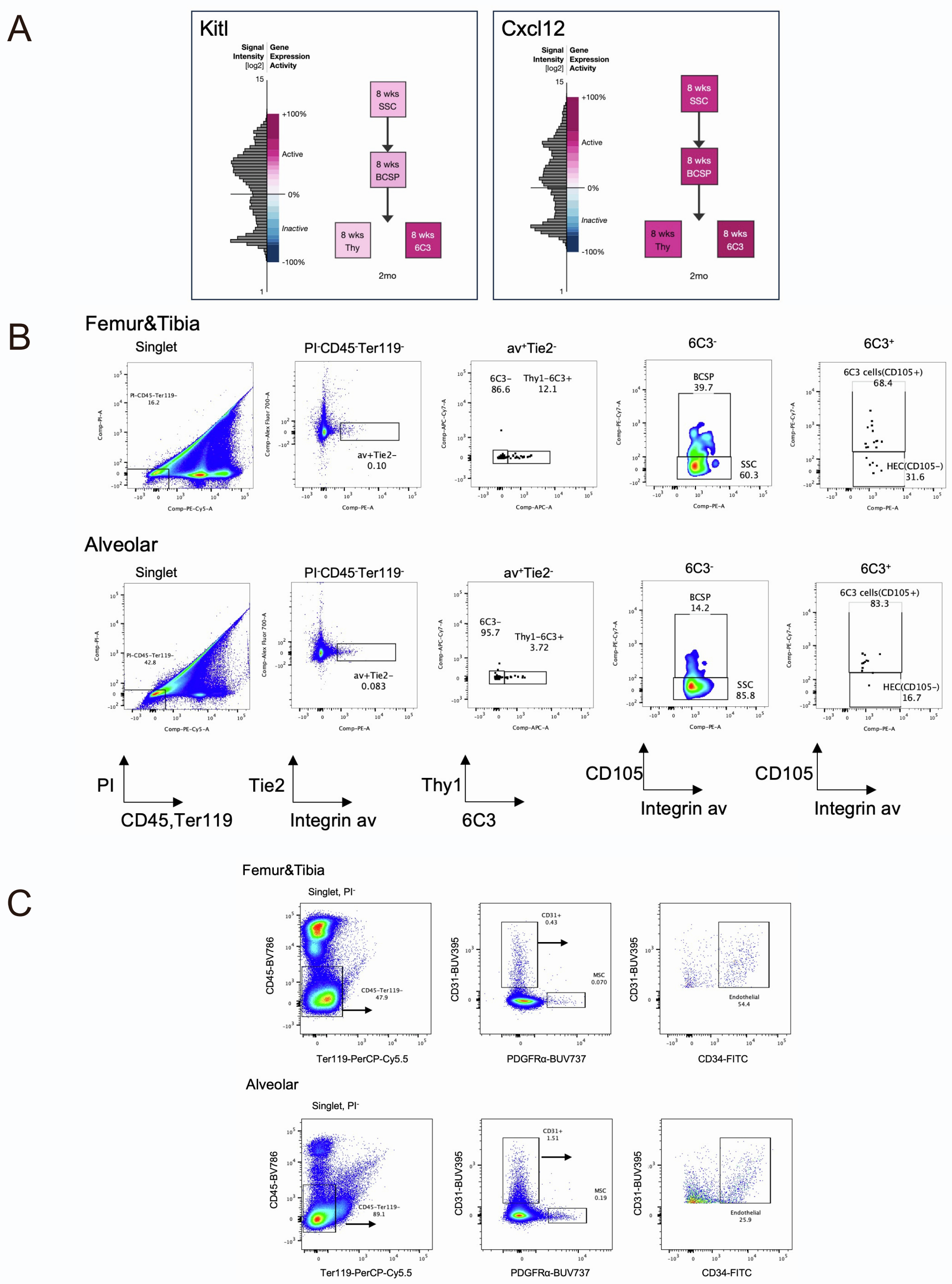

Figure S4 Expression and Identification of Skeletal, Mesenchymal, and Endothelial Cell Populations in Alveolar and Femur/Tibia Bone Marrow

(A) Expression of *Kitl* and *Cxcl12 in skeletal stem cell lineages*

Relative expression of *Kitl* and *Cxcl12* in skeletal stem cell lineages of 8-week old mouse as determined by Affymetrix Mouse Genome 430 2.0 Array analysis. Pink represents increased expression, blue represents decreased expression.

(B) Identification of Skeletal Stem and Progenitor Cell Populations in Alveolar and Femur/Tibia Bone Marrow. Representative flow cytometry plots for the identification of skeletal stem cells (SSC; CD45^{-}Ter-119^{-}Tie2^{-}AlphaV^{+}Thy^{-}6C3^{-}CD105^{-}), bone, cartilage, stromal progenitors (BCSP; CD45^{-}Ter-119^{-}Tie2^{-}AlphaV^{+}Thy^{-}6C3^{-}CD105^{+}), Thy subpopulation (CD45^{-}Ter-119^{-}Tie2^{-}AlphaV^{+}Thy^{+}6C3–CD105^{+}), and 6C3 subpopulation (CD45^{-}Ter-119^{-}AlphaV^{+}Thy^{-}6C3^{+}CD105^{+}) in ftp-BM and al-BM.

(C)Analysis of Mesenchymal Stromal and Endothelial Cells in Alveolar and Femur/Tibia Bone Marrow. Flow cytometry plots showing the identification of mesenchymal stromal cells (MSC; CD45^{-}Ter-119^{-}CD31^{-}PDGFRα^{+}), and endothelial cells (CD45^{-}Ter-119^{-}CD31^{+}CD34^{+}) in ft-BM and al-BM.
